# Melatonin at Crossroads with Phytohormones: Interactions Under High Light Stress

**DOI:** 10.3390/ijms262110531

**Published:** 2025-10-29

**Authors:** Ivan A. Bychkov, Natalia V. Kudryakova, Victoria V. Shitikova, Victor V. Kusnetsov

**Affiliations:** K.A. Timiryazev Institute of Plant Physiology RAS, 35 Botanicheskaya St., 127276 Moscow, Russia; ivan.a.b@mail.ru (I.A.B.); vicry@yandex.ru (V.V.S.); vkusnetsov2001@mail.ru (V.V.K.)

**Keywords:** *Arabidopsis thaliana*, high light stress, melatonin, mRNA sequencing, phytohormones

## Abstract

Melatonin (MT), an antioxidant and growth regulator, interacts with almost all phytohormones, but the molecular mechanisms of these interactions are poorly understood. Using mRNA sequencing (mRNA-seq) technology, we analysed the global regulation of MT-induced expression of genes involved in metabolism, signalling and responses to major phytohormones under prolonged high-intensity light (HL) stress. Plants respond to MT through the activation of auxin and brassinosteroid (BS) response genes, which were identified among the enriched categories of differentially expressed genes (DEGs) with increased expression, and the suppression of abscisic acid and ethylene signalling and response genes, which were among the enriched downregulated categories. MT also suppressed growth-inhibiting genes involved in jasmonic acid (JA) and salicylic acid (SA) signalling and response and activated genes encoding the growth-promoting hormones gibberellins and cytokinins (CKs), which is consistent with the role of MT in stress alleviation. However, the expression of some unique genes, which are positively or negatively modulated by stress, was reinforced by MT treatment, illustrating the extraordinary type of regulation that enhances the action of specific hormone-mediated mechanisms. The study of signal integration between MT and hormones with the involvement of signalling mutants revealed that some interactions are regulated at the transcriptional level and require the activity of relevant signalling pathways. Disruption of *CAND2* completely abolished melatonin-dependent activation of the mitogen-activated protein kinases MAP3K17 and MKK7, suggesting that the MAP3K17-MKK7 module is an important player in the MT-triggered MAPK pathway, acting downstream of CAND2.

## 1. Introduction

Melatonin, a pleiotropic molecule with a variety of functions in reactive oxygen species (ROS) scavenging and plant signalling pathways, has been the target of numerous studies given its dual role in plant responses to internal and external challenges. Its ability to detoxify ROS molecules as toxic byproducts of aerobic metabolism is based on its capacity to bind them, reducing the risk of ROS injury. However, similar to ROS, melatonin may act as a signalling molecule necessary for the progression of basic biological processes. In the second state, melatonin meets all the requirements for phytohormones, since it acts at low concentrations, is perceived through the receptor and transported through the xylem, has a variety of functional activities and is integrated into the phytohormone network [1,2].

The pathways of melatonin synthesis in plants are well known. They include two main stages: the synthesis of serotonin (5-hydroxytryptamine) via the conversion of tryptophan into tyramine by tryptophan decarboxylase (TDC) followed by the formation of serotonin by tryptamine-5-hydroxylase (T5H) and the synthesis of melatonin from serotonin. The second stage is also a two-step reaction involving three distinct enzymes (SNAT, ASMT, and COMT) and two independent pathways [3]. The catabolism of melatonin is carried out enzymatically by several melatonin hydroxylases, such as M2H and M3H [4]. Melatonin is perceived via the PMTR1/CAND2 receptor, triggering G protein signalling and activating MAPK cascades [5].

While melatonin, with its reasonably acquired status as an essential phytohormone, clearly plays a role in interactions with almost all known plant hormones, the molecular mechanisms underlying such links remain mostly vague. Many of the links are simply correlative, at most demonstrating the ability of melatonin to alter hormone levels and the expression of hormone synthesis or signalling genes. In general, phytohormones and MT, as components of the plant hormone system, are assumed to interact at the level of transcription of synthesis and signalling genes, regulating physiological processes and stress responses, although interactions at the protein level may also exist. Extensive crosstalk between MT and hormones at the transcriptional level has been shown for growth-promoting hormones, namely, auxins [6], cytokinins [7,8], gibberellins [9] and brassinosteroids [10,11,12], as well as for growth-retarding hormones, including abscisic acid [9], jasmonates [13], salicylic acid [14], and ethylene [15]. MT and hormones can exhibit both synergistic and antagonistic effects by regulating each other’s transcriptional activity. The combined application of melatonin with different hormones has been proven to be more effective than the use of melatonin alone [16].

The identification of melatonin-responsive genes on the basis of transcriptomic data revealed that melatonin altered gene expression along stress-induced hormone signalling pathways [17]. While the majority of auxin-responsive genes were downregulated in response to 1 mM of melatonin, most genes in the ABA, SA, JA and ET pathways were upregulated, confirming the critical role of melatonin in defense against both biotic and abiotic stresses in plants. However, Weeda et al. used high concentrations of melatonin (1 mM), which is many times higher than physiological levels that underlie melatonin signalling and its crosstalk with other phytohormonal pathways. Conversely, Wan et al. [18], via transcriptome analysis of MT-treated seedlings, reported that moderate concentrations of melatonin and serotonin (10 and 50 µM) do not affect the abundance of the auxin transporters AUXI and PIN1/2/4/7 or auxin accumulation. The functional versatility of MT and its ability to act at very high and low concentrations suggest the existence of various binding sites that require different saturation concentrations [19]. However, whether the hormonal activity of MT is realized at high concentrations remains debatable.

As a consequence of the changes mediated by melatonin, one should expect considerable transcriptome changes including the regulation of phytohormone biosynthesis and degradation as well as the regulation of hormone receptors and signalling. However, the role of melatonin as a modulating agent remains controversial, since most hormonal responses in plants target different members of gene families depending on specific input pathways. Therefore, global expression studies simultaneously covering the regulation of all major plant hormonal systems within the same experimental set up might help in elaborating new concepts in plant-hormone interactions. One of the most interesting aspects of this interplay is the exact players underlying the crosstalk between melatonin and other hormones. The experiments presented in this article were performed to investigate at least some of these issues.

In this study, we used physiological concentrations of MT to identify potential MT targets involved in hormonal pathways. Since the functions of melatonin as a regulator of intracellular processes are clearly manifested under conditions of oxidative stress, we analysed the results of comparative transcriptome profiling of melatonin- and mock-treated *Arabidopsis* plants exposed to high light, which was superimposed on 10-fold lower light conditions under growth conditions.

Using RNA-seq analysis, we identified more than 70 melatonin-regulated genes encoding hormone-related proteins with diverse functions involved in specific plant responses under persistent stress conditions. In addition, we provide evidence that at least some melatonin–hormone interactions are regulated at the transcriptional level and depend on the MT receptor CAND2. Finally, we showed that melatonin-mediated regulation of hormonal genes may require the activity of relevant signalling pathways, in addition to noncanonical signal integration mechanisms.

## 2. Results

### 2.1. Melatonin Triggers Protective Physiological Responses Under Excess Light

The ability of exogenous protectants to exert beneficial effects on plants under stress conditions is commonly evaluated on the basis of their ability to ameliorate physiological indicators. It has been postulated that prolonged exposure to high irradiance causes increased ROS levels, the damaging effects of which include the oxidation of lipids, proteins, and enzymes necessary for proper function of the cell [20]. Increased production of ROS limits the ability of a plant to utilize light energy through photosynthesis and results in an increase in the levels of enzymatic and nonenzymatic scavengers.

**Figure 1:**
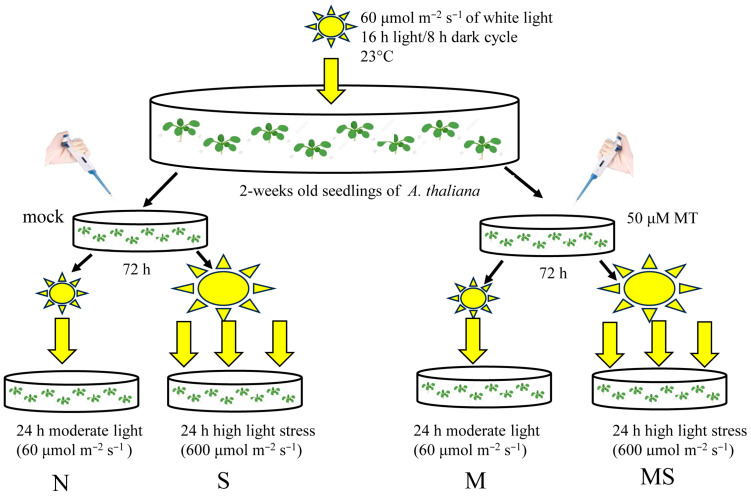
Experimental design. N—control, S—stress, M—melatonin, MS—melatonin + stress.

To assess the effectiveness of MT treatment on photoprotective mechanisms, *Arabidopsis* plants were pretreated for 72 h with 50 μM of melatonin or an equal aliquot of ethanol (control). They were then transferred under an HPI-T2 2000 W/646 lamp (Philips, The Netherlands) with a luminous energy flux of 600 μmol m^−2^ s^−1^ and exposed to continuous high light irradiation for 24 h (Figure 1). The spectra of lamps used under normal growing conditions and excess light were similarly based on information provided by the lamp manufacturers (Supplemental Figure S1). To ensure uniform illumination, plates with experimental plants were placed in a narrow focal zone of the lamp and rotated several times during the experiment.

**Figure 2:**
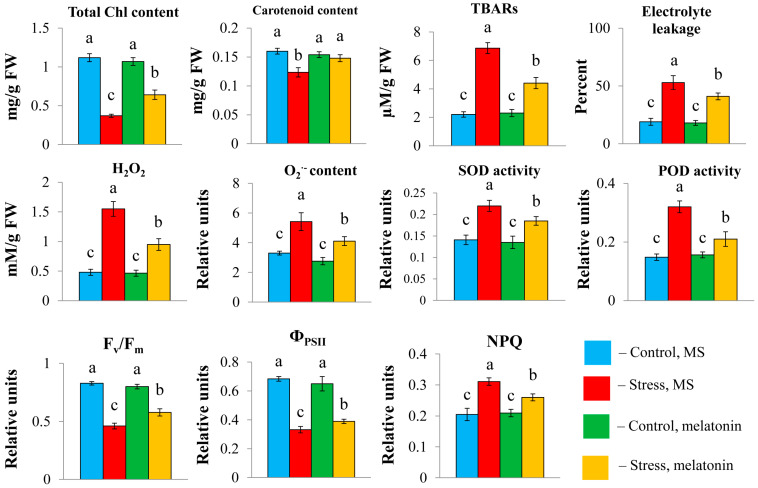
Physiological parameters of *Arabidopsis* plants subjected to high light stress and melatonin treatment. Two-week-old plants were shifted for 72 h to paper filters moistened with liquid MS media supplemented or not supplemented with 50 μM of melatonin and exposed to HL stress (PPFD 600 μmol m^−2^ s^−1^) for 24 h. Control plants were grown under moderate light (PPFD 60 μmol m^−2^ s^−1^. The data presented are the mean values ± SEss (*n* ≥ 3). Different letters denote statistically significant differences at *p* < 0.05 (ANOVA with post hoc Tukey’s multiple-comparison test).

Oxidative stress triggered by long-term high light contributed to a substantial decrease in the total carotenoid and chlorophyll (a + b) contents, which were partly restored following the combined application of HL and melatonin (Figure 2). Plants exhibited significant damage to their plasma membranes under HL, as evidenced by multiple increases in the levels of lipid peroxidation products and increased electrolyte leakage. Hydrogen peroxide and OH^−^ levels were also elevated. However, the exposure of the plants to HL + MT resulted in a substantial decrease in these indicators. Furthermore, the lower vulnerability of plants to stress in the presence of melatonin was indicated by the activities of the enzymatic scavengers superoxide dismutase (SOD) and peroxidase, which were significantly lower than those under HL.

Melatonin treatment significantly reduced photoinhibition, as evidenced by increases in the Fv/Fm ratio and ΦPSII (Figure 2). With the addition of melatonin, the value of nonphotochemical chlorophyll fluorescence quenching (NPQ), a major factor in the regulation of light harvesting and protection of PSII reaction centers, decreased compared with that under stress. These findings suggest the efficient ability of MT to remove excess ROS in chloroplasts to balance redox reactions and that the photosynthetic system functions in cooperation with the mitochondrial respiratory chain.

Taken together, these results show that melatonin effectively mitigates stress responses induced by excess radiation, which in turn may depend on hormonal pathways that integrate information from internal and external signals. Elucidating the molecular events underlying these massive changes in plant physiology is key to understanding the fundamentals of melatonin function.

### 2.2. Transcriptional Regulation of Hormone-Related MT Targets Under HL Stress

Transcriptome sequencing was used to investigate global changes in the gene expression of melatonin-treated plants exposed to excess light. Thirty-three million Illumina reads, were obtained for each sample and 97% passed the quality check. A total of 88–91% of the reads were uniquely mapped to the *Arabidopsis thaliana* reference genome, indicating that the sequencing data were sufficiently aligned for further analysis [21].

A total of 5542 differentially expressed genes (DEGs) were identified under HL stress, and 4910 DEGs were identified under combined treatment with melatonin and HL, among which 4131 DEGs were common (Supplemental Tables S1–S3). Notably, under moderate light, the transcriptional response of *Arabidopsis* to long-term melatonin treatment was significantly weaker and included only 49 DEGs. Some of them could also be affected by HL treatment or combined HL + M treatment.

To delineate effects specifically due to MT and those induced by HL treatment, we selected 256 genes that showed a twofold change in the HL+MT-treated samples compared to their values in the HL-treated samples. These 256 genes appeared to be core MT-responsive genes under long-term MT treatment. Seventy-nine of these genes were classified as hormone signalling and metabolic genes or hormone response genes.

**Figure 3:**
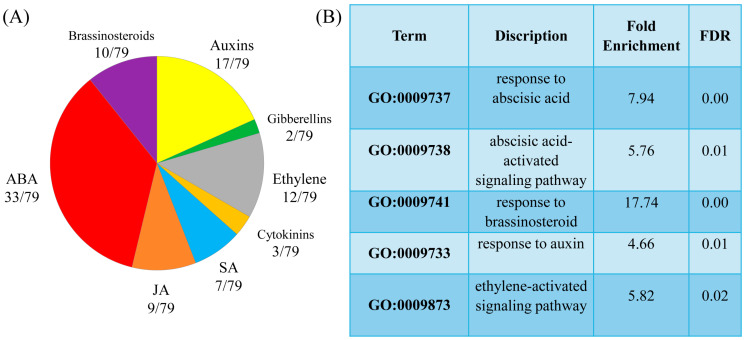
(**A**)—Pie chart showing the distribution of hormone-responsive genes affected by melatonin under long-term high light stress. Several MT target genes respond to signals from more than one hormone. (**B**)—GO terms enriched among hormone-responsive genes with *p* ≤ 0.05.

The GOTERM_BP _DIRECT classification of the DEGs according to their biological functions revealed auxin and brassinosteroid response genes among the upregulated enriched categories and ethylene and abscisic acid (ABA) signalling and response genes among the enriched downregulated categories. In addition, melatonin modulated the expression of genes involved in signalling, metabolism, and response to jasmonic acid (JA), salicylic acid (SA), and ethylene (Figure 3). Furthermore, there was a marked decrease in the expression of several BS (brassinosteroid)-responsive genes.

Some MT-regulated genes respond to multiple hormone signals, such as *BT2* (*AT3G48360*), which is involved in the response to ABA, SA, JA and auxin, or *Thi2.1* (thionin 2.1), regulated by ethylene, SA and JA. The final expression vector of such genes is apparently determined by the hormone balance, which is dependent on external and internal stimuli. In general, MT contributed to the suppression of hormonal pathways activated by stress and induced pathways associated with growth processes. However, opposite patterns of regulation were shown for certain genes. Below, we will consider in detail some aspects of melatonin regulation of hormone-dependent genes under excess light.

#### 2.2.1. ABA-Related Genes

The involvement of melatonin in the regulation of ABA-responsive genes has been shown in a number of studies, indicating that the crosstalk between MT and ABA is highly variable. In general, melatonin induces the downregulation of ABA biosynthesis genes and the upregulation of ABA catabolism genes, resulting in a decrease in ABA levels and signalling. However, the opposite regulation has also been identified [2,22].

**Figure 4:**
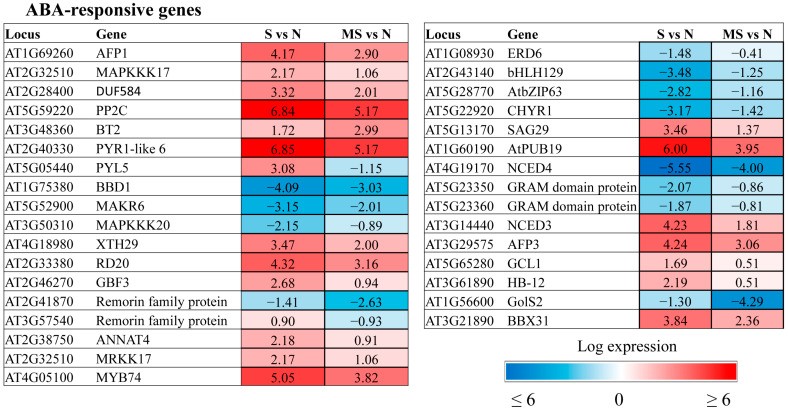
Heatmaps of ABA-responsive genes that are affected by melatonin under high light stress, with changes in expression levels of at least 2-fold compared with stress-induced levels.

The transcriptomic data provided a list of ABA-affected genes that were specifically regulated by MT under HL conditions (Figure 4). As anticipated, they comprised a number of genes belonging to the abscisic acid-activated signalling pathway (GO:0009738), including receptors (*AT2G40330 PYR1-like 6*, *AT5G05440 PYL5*), kinases (*MAPKKK17*,*18*, *MAKR6*) and transcription factors. We suggest that melatonin and ABA converge on these specific sets of transcriptional targets. One such gene, *GCR2-like 1* (*AT5G65280*), encodes a protein with reported similarity to GCR2, a putative G protein-coupled receptor that is thought to be an ABA receptor. Recently, it was proposed that GCR2 functions as an intracellular modulator of ABA signal transduction or ABA metabolism and/or as a modulator of G protein signalling [23]. Given the involvement of G proteins in melatonin perception, further study of the role of GCL1 is tempting.

Some ABA signalling genes were in parallel classified into the category response to abscisic acid (GO:0009737). This category included genes involved in various ABA-dependent functions, such as Ca signalling, senescence, sucrose transport, and ubiquitination. Most of them exhibited a mitigating effect of MT on stress-induced expression changes. These genes were either downregulated by MT when activated by HL stress or upregulated when excess light caused their repression. However, changes in the expression of certain genes, such as the remorin family gene *AtREM4.2* (*AT2G41870*), in response to stress were further reinforced when these genes were combined with melatonin treatment, indicating their involvement in specific MT-driven mechanisms.

#### 2.2.2. Ethylene-Related Genes

**Figure 5:**
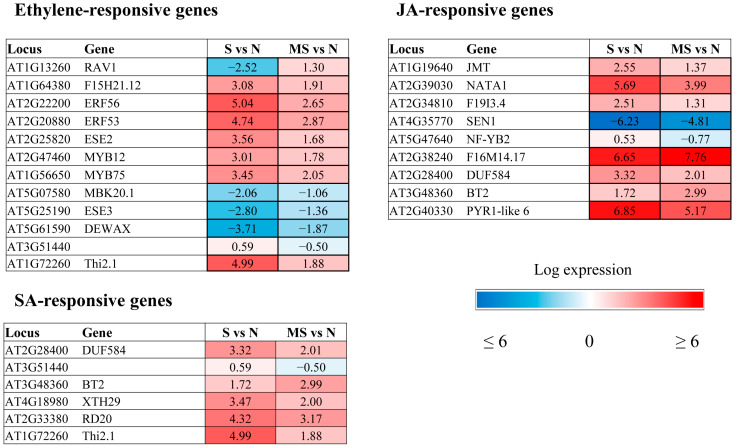
Heatmaps of ethylene-, SA- and Ja-responsive genes that are affected by melatonin under high light stress, with changes in expression levels of at least 2-fold compared with stress-induced levels.

The major trends in MT regulation of ethylene-related genes are largely similar to those shown for ABA-associated genes (Figure 5). The expression of ethylene-modulated *PAP1/MYB75* (producing anthocyanin pigments) and *MYB12*, which are involved in the flavonoid biosynthetic pathway, was elevated in response to photooxidative stress, in accordance with the photoprotective effect of anthocyanins, which enhance photoprotection by filtering or reflecting harmful radiation and removing various types of ROS through aromatic hydroxyl and orthodihydroxyl groups [24]. Treatment with melatonin, a stress-alleviating agent, reduced the expression of these transcriptional regulators, thereby allowing plants to adapt to changing stress conditions.

MT also contributed to the downregulation of ERF/AP2 transcription factor family genes belonging to the DREB subfamily, whose expression was induced by HL stress. In contrast, MT mitigated the downregulation of two ERF/AP2 family genes, *DEWAX* (*AT5G61590*) and *DEWAX2* (*AT5G07580*). *DEWAX2* was shown to function negatively in the transcriptional regulation of cuticular wax biosynthesis in *Arabidopsis* [25]. Therefore, its suppression during stress increases wax synthesis, which is involved in limiting nonstomatal water loss, protecting against UV radiation, and defending against pests and pathogens. Melatonin, which reduces the effects of stress, promotes an increase in its expression, leading to a subsequent reduction in the total wax load and an increase in the cuticular transpiration rate. Thus, direct targets of MT may be cell wall component genes and their regulators.

Another plant cell wall-associated MT target is the ethylene response factor gene *ESE3* (*AT5G25190*), which is coregulated with *AGP17* and *18* encoding arabinogalactan proteins (AGPs) in *Arabidopsis*. AGPs may serve as cell wall integrity sensors and play a chaperoning role in living plant cells, guiding cell wall assembly and hence cell expansion and development [26].

#### 2.2.3. JA- and SA-Related Genes

On the basis of the data reported thus far, melatonin suppresses the action of JAs by inhibiting their synthesis and the synthesis of JAZ proteins [27]. Accordingly, MT treatment contributed to the downregulation of 4 genes (*JMT*, *NATA1*, *AT2G34810* and *NF-YB)* involved in the response to jasmonic acid (Figure 5).

The negative effect of MT on JA levels may also be inferred from the upregulation of *JOX4* (*At2g38240*), encoding JOX oxygenase, an enzyme that hydroxylates JA to 12-OH-JA, the inactive form of JA [28]. *JOX4* gene expression, which was dramatically upregulated under HL stress (6.65 log2), increased further (7.77 log2) under HL + MT treatment. We thus conclude that MT may function by reducing JA accumulation and the expression of JA-responsive genes, presumably to minimize plant growth inhibition associated with JA-mediated defense responses.

An examination of the impact of exogenous MT on stress-induced expression suggested that MT may synchronously influence SA- and ethylene-associated genes, in addition to JA-mediated effects. *Thi2.1* (*thionin 2.1*, *AT1G72260*), an established marker gene for JA signalling, and *SSL6* (AT3G51440), encoding a member of the strictosidine synthase-like gene family, are also implicated in the response to salicylic acid and ethylene [29,30]. Both genes were shown to be induced by stress and are likely employed in plant defense mechanisms, which require the synergistic involvement of several hormones.

#### 2.2.4. Auxin-Related Genes

The similar effects of auxin and melatonin on a range of processes, as well as the presence of a common precursor and structural similarity, have often been treated as evidence for the functional identity of these two regulators. However, several studies provide strong arguments in favour of the auxin-independent pathways of MT activity. In our tests, at least 15 genes classified into the “response to auxin” and/or auxin-activated signalling pathway categories were significantly regulated by MT.

**Figure 6:**
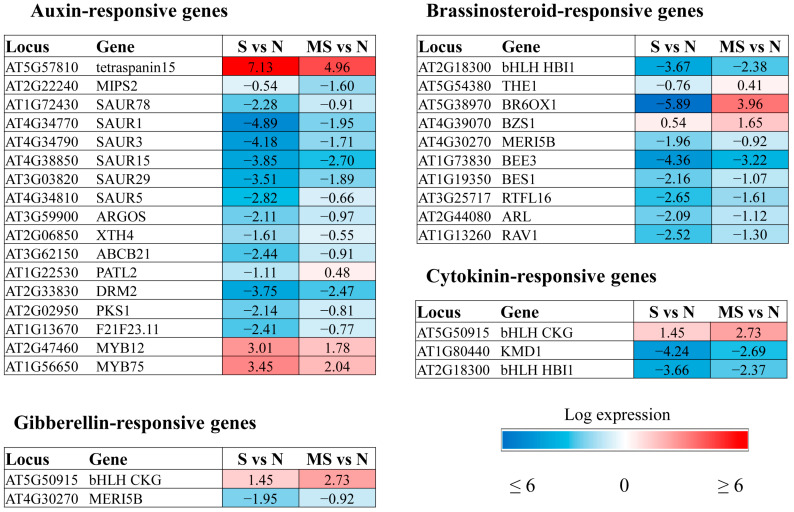
Heatmaps of auxin-, brassinosteroid-, cytokinin- and GA_3_-responsive genes that are affected by melatonin under high light stress, with changes in expression levels of at least 2-fold compared with stress-induced levels.

We detected a strong effect of MT treatment on the expression of *SMALL AUXINE UP RNA (SAUR)* genes (Figure 6). *SAURs* constitute the largest family of early auxin response genes involved in a wide range of biological processes [31]. Various members of the SAUR family can act as antagonists, being activated or repressed in response to growth-promoting or stress-related hormones. Almost all SAUR genes classified as MT targets in our tests were previously shown to be activated by brassinosteroids and partly by gibberellins, in addition to auxin [31].

Among other MT-targeted genes are *PATL2* (*PATELLIN2*, *AT1G22530*), involved in auxin-mediated PIN1 relocation and plant development [32], and *ABCB21* (*P-glycoprotein 21*, *AT3G62150*), which encodes a facultative transporter that controls auxin concentrations in plant cells [33]. Auxin redistribution, which modulates phototropism and leaf positioning under HL, is also regulated by phytochrome kinase substrate (PKS) family proteins, which are required either for the establishment of a local lateral auxin gradient or for the response to this gradient [34]. Melatonin upregulated *PKS1* (*phytochrome kinase substrate 1 AT2G02950*), whose expression was suppressed under HL, contributing to photoprotection via the regulation of *phot1* and *phot2* under high light conditions.

Other MT targets responsive to auxins include *ARGOS* (*AT3G59900*), an auxin-regulated gene involved in cell proliferation and organ growth [35]; *DAP2* (*Dormancy/auxin-associated family protein AT2G33830*), a negative regulator of basal defence against virulent bacterial pathogens [36]; and *AtXTH4* (xyloglucan endotransglucosylase hydrolase 4, *AT2G06850*). The latter gene was proposed to stimulate xylem cell production and modulate secondary wall thickening [37].

Melatonin mitigated stress-induced alterations in the expression of all the aforementioned auxin-related genes (Figure 6). The only exception was *MIPS2* (*AT2G22240*), encoding *myo*-inositol-1-phosphate synthase 2, which was further suppressed by HL + MT. Melatonin-dependent suppression of *MIPS2* may contribute to inositol homeostasis and stress resistance via crosstalk with the *my*o-inositol pathway.

#### 2.2.5. Brassinosteroid-Related Genes

BR is a growth-promoting hormone that exhibits extensive crosstalk at the transcriptional level with almost all known hormones to regulate many shared target genes [38]. Among the melatonin target genes associated with the brassinosteroid response (GO:0009741), we found ABA-responsive *RAV1*, gibberellin-responsive *XTH24* and several auxin-regulated genes, such as *SAURs*, *ARGOS-like protein* (*AT2G44080*) and *ROTUNDIFOLIA-like* (*AT3G25717*), as well as *AT2G18300*, which encodes a basic helix–loop–helix (bHLH) DNA-binding protein (Figure 6). In addition to BR, *AT2G18300* is also regulated by cytokinin and IAA. Since these genes are predominantly required for cell expansion during vegetative growth, they were downregulated under stress and partially restored by MT treatment.

The same was observed for genes encoding components of BS signalling and the synthesis of *BEE3* and *BR6OX1*. We propose that BR deficiency contributes to increased stress tolerance under HL conditions and the downregulation of BR-induced genes involved in cell elongation.

In contrast to the decreased expression of BS-related genes under stress conditions, *BZS1* (*AT4G39070*), a brassinosteroid-regulated *BZR1* target gene, was slightly upregulated by HL and further induced by MT. *BZS1* was shown to be a negative regulator of cell elongation during hypocotyl growth [39]; hence, its upregulation by MT contradicts the general trend of MT-promoted cell expansion. It is tempting to speculate that BZS1, a plasma membrane-associated protein, may also play a role in activating cellular communication by influencing protein–protein interactions.

#### 2.2.6. Cytokinin-Related Genes

Transcriptomic studies revealed few CK-associated genes whose expression was significantly (more than 2-fold) modulated by MT under HL conditions (Figure 6). One of them is *CKG*, which encodes a basic helix–loop–helix (bHLH) DNA-binding superfamily protein. *CKG* was upregulated by HL stress and further activated by combined MT + HL treatment. The downstream targets of CKG are mainly involved in CK-mediated regulation of cell size and cell cycle progression, promoting growth, early flowering, and increased productivity [40].

The opposite regulatory pattern was revealed for *AtKFB20* (*AT1G80440*). *AtKFB20* (also called *KISS ME DEADLY*) encodes a member of a family of F-box proteins that act as components of the canonical SCF-type protein-ubiquitin ligase complex family. *AtKFB20* selectively targets CK type-B response regulators for degradation and is involved in the negative regulation of the cytokinin response [41]. It has been suggested that the downregulation of CK signalling is one of the mechanisms used by plants to adapt to adverse conditions [42]. Therefore, the downregulation of *AtKFB20* expression by MT can be interpreted in terms of stress mitigation.

### 2.3. Validation of Hormone-Related Genes Identified by RNA-Seq Analysis

**Figure 7:**
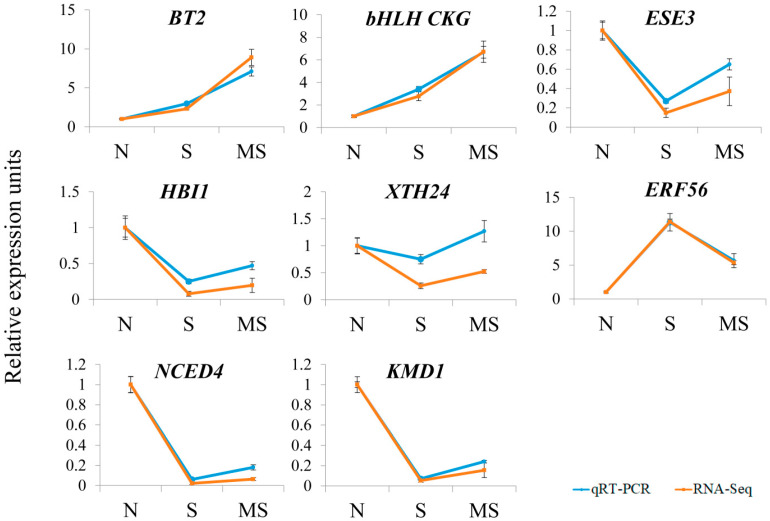
Validation of the mRNA-seq data via qRT–PCR. N, moderate light; S, high light; MS, melatonin + high light. qRT–PCR was performed on the same samples used for the RNA-seq experiments.

We validated the RNA-Seq data via quantitative RT–PCR, which allows independent assessment of transcript levels. For this purpose, we examined the expression of 8 genes up- or downregulated by MT treatment via the same RNA probes used for the RNA-Seq analysis. We chose genes related to hormone signalling, synthesis, and response and representing all major groups of phytohormones. The RT–PCR data were mostly consistent with the RNA–Seq results, confirming the accuracy and reliability of the transcriptome profiling results obtained via RNA–Seq (Figure 7).

### 2.4. Selective Involvement of CAND2 in the MT-Related Responses of Hormone Genes

Identification of the players involved in the crosstalk between MTs and hormones necessarily requires an analysis of the roles of MT receptors. Recently, we showed that the network of ABA-related genes under HL, at least in part, depends on the MT receptor CAND2 (AtPMTR1/AtCAND2). In particular, the ABA signalling genes *ABI1*, *2*, and *5* do not respond significantly to MT treatment in the melatonin receptor mutant *cand2* [23], whereas *ABI3* and *4* are affected by MT, both in the WT and the mutant, demonstrating the specificity of transcriptional responses.

To test whether disrupting the normal perception of melatonin may also result in the loss of ability to influence the expression of other hormone-associated genes, we used a *cand 2-1* mutant (Salk_071302), which has T-DNA inserted in the promoter region. In accordance with the experimental design used for RNA-Seq analysis, the mutants pretreated with 50 μM of melatonin or an equal aliquot of ethanol were transferred under HL (600 μmol m^−2^ s^−1^ for 24 h) and further examined via qRT–PCR.

**Figure 8:**
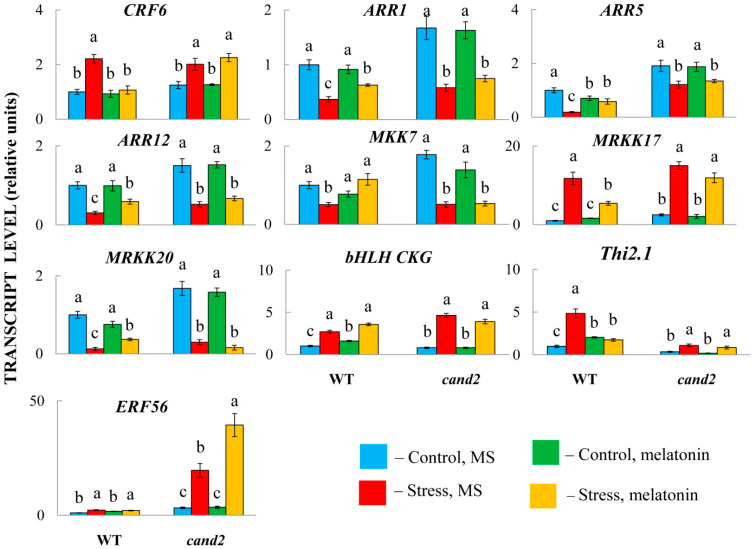
Expression levels of selected hormone-responsive genes in the WT and *cand2* backgrounds after treatment with HS or MT. The data presented are the mean values ± SEss (n ≥ 3). Different letters denote statistically significant differences at *p* < 0.05 (ANOVA with post hoc Tukey’s multiple-comparison test).

The analysis of the response of the mutants to MT treatment revealed that the expression of the genes under study was partially dependent on CAND2 (Figure 8). Thus, in WT plants, the transcript levels of the CK signalling genes *ARR1*, *5*, and *12* increased, whereas the levels of *CRF6* decreased compared with those in untreated stressed plants but remained unchanged in the melatonin receptor mutant. Likewise, ABA-responsive *MRKK17*, *MRKK20* and *MTT7*, encoding MAP kinases, were unaffected by MT treatment in the mutants, indicating that their transcriptional regulation is achieved through the receptor CAND2. Similar results were shown for *CKG* (*AT5G50915*), encoding a bHLH superfamily DNA-binding protein, and *Thi2.1* (*AT1G72260*), encoding a cysteine-rich protein that is expressed in response to a variety of pathogens.

Nonetheless, the MT-induced responses of some genes, particularly the ethylene-responsive transcription factor *ERF56* and the aforementioned *ABI3* and *ABI4*, were independent of CAND2, as the mutants presented similar expression patterns under both HL and the combination of HL and MT treatment. These findings may indicate that the activity of unknown/undefined MT receptors is required for the transcriptional regulation of these genes. On the other hand, nontranscriptional signalling through the direct interaction of signalling proteins may also explain the independence of MT action from the receptor CAND2.

### 2.5. The Role of Hormone Signalling in MT-Triggered Responses

We further asked whether the signal integration of MT and hormones necessarily requires the transcriptional response of hormone signalling genes. To test this hypothesis, we employed several *Arabidopsis* mutants with impaired signal transduction and their parental forms. Two mutants, *abi1-1* and *etr1*, were compromised in the perception of ABA and ethylene. The other two genes, *bri1-6* and *ahk2/3*, were disrupted in genes encoding brassinosteroid and cytokinin receptors. ABA and ethylene are hormones that are primarily associated with stress, whereas BS and CK are growth-promoting phytohormones that regulate various physiological processes and development.

**Figure 9:**
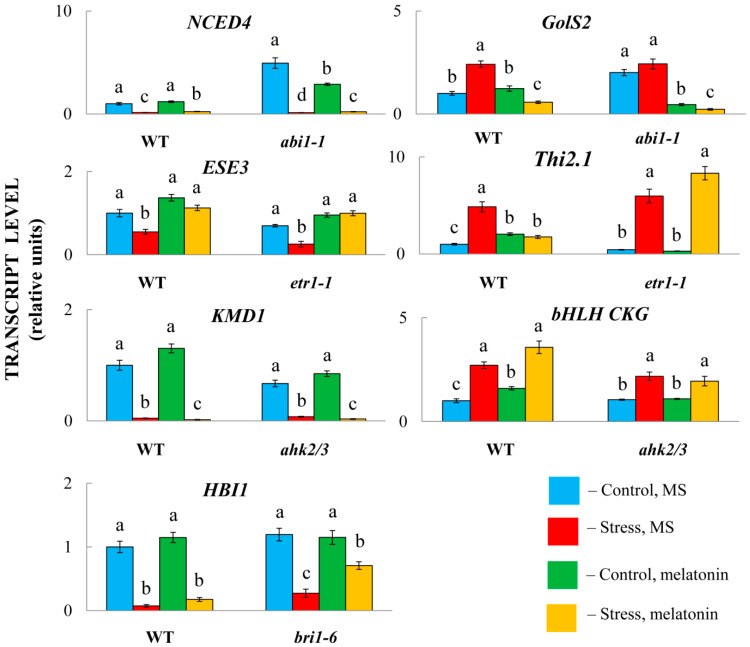
Expression levels of hormone-responsive genes in the WT and phytohormone receptor mutants after treatment with HS or MT. The data presented are the mean values ± SEss (n ≥ 3). Different letters denote statistically significant differences at *p* < 0.05 (ANOVA with post hoc Tukey’s multiple-comparison test).

While melatonin significantly modulated the expression of selected genes in the wild-type plants, the response of the mutants varied (Figure 9). In particular, in combination with HL, MT positively regulated the accumulation of transcripts of the ABA catabolism gene *NCED4* and negatively regulated the ABA response gene *GolS2* compared with stress-induced levels in both LERs and *abi1-1*. Similarly, melatonin had no apparent difference in modulating the expression of the ethylene marker gene *ESE3* (*AT5G25190*) in *etr1-1* compared with Col0 or *AT1G80440* in *ahk2/3*, which encodes a protein from a KISS ME DEADLY (KMD) family that targets type-B ARR proteins for degradation. Similar *bri1* receptor-independent expression was found for *HBI1*, a positive regulator of BR-triggered responses [43]. These results imply that melatonin-mediated regulation of these hormonal genes does not necessarily depend on the transcriptional regulation of hormone signalling.

In contrast, melatonin positively regulated the expression of the gene encoding major helix-loop-helix (bHLH) superfamily DNA-binding protein (*AT5G50915*, *CKG*) in Col0, whereas no increase in expression was observed in the *ahk2/3* mutant. Furthermore, *Thi2.1* (*AT1G72260*), which is induced by ethylene and jasmonic acid, did not respond to MT treatment in *etr1-1*. Taken together, these findings suggest that melatonin-mediated regulation of some hormonal genes requires the activity of relevant signalling pathways.

## 3. Discussion

The opportunity to examine the relationships between melatonin and plant hormones under HL stress via global RNA-seq analysis has contributed to the identification of many new MT target genes involved in hormone-related networks. These genes include genes associated with changes in hormone levels and signalling, as well as hormone-responsive genes implicated in specific stress-related reactions. However, the responses of certain genes were reinforced by MT treatment, suggesting specific mechanisms of melatonin action.

In general, light-driven stress responses are mitigated in MT-treated plants, as determined by changes in the activities of target genes and physiological indicators. In particular melatonin treatment contributes to maintenance of membrane integrity as evidenced by lower electrolyte leakage and the levels of lipid peroxidation products. Furthermore, exogenous melatonin decreased accumulation of ROS preventing chloroplast damage and reducing photoinhibition. Elevated HL tolerance in *Arabidopsis* is also confirmed by the decreased activity of ROS-responsive antioxidant enzymes. In our previous study [44], we showed that leaf respiration, which was significantly increased under HL stress through both cytochrome (CP) and alternative oxidation (AO) pathways, was reduced by MT treatment, primarily through inhibition of CP and, to a lesser extent, AO. It should be noted that the moderate stress (600 μmol m^−2^ s^−1^) used in the experiment, superimposed on 10-fold lower light conditions under growth conditions (60 μmol m^−2^ s^−1^), promotes significant physiological damage rather than cell death as in the case of severe light stress (>1000–2000 μmol m^−2^ s^−1^), and is often considered as part of the acclimation response that increases tolerance to severe light stress.

Acclimation to HL also depends on hormonal signals. Consistent with the results of Huang et al. [45], plants respond to HL by dynamically upregulating ABA-, JA-, ethylene- and SA-related genes, which are involved in light acclimation. Concurrently, genes involved in hormones related to plant growth and development, such as BRs, auxins, and cytokinins, presented repressed expression. The downregulation of these genes correlates with the observation that plant growth is retarded under HL stress. In our tests, melatonin-modulated gene expression was aimed mainly at alleviating the HL stress-induced response. As a result of MT treatment, growth-inhibiting genes were largely suppressed, while growth-promoting genes were upregulated compared with their stress-induced levels.

To a certain extent, these data contradict the findings of Weeda et al. [17], according to which most genes in the ABA, SA, JA and ET pathways were upregulated, whereas the majority of auxin-responsive genes were downregulated in response to 1 mM of melatonin. These discrepancies indicate that plants sense 1 mM of melatonin as stress. Notably, at low levels (100 pM) of melatonin, far fewer genes were affected. Only some of these genes are associated with hormone responses. Moreover, not all genes up- or downregulated by low melatonin (100 pM) were similarly regulated by high (1 mM) melatonin, confirming that melatonin may play significantly different roles under low and high concentrations. The way in which melatonin performs its numerous functions depends largely on the plant system being examined, as well as on the nature of the stress factors.

This study presents a comprehensive comparison of the effects of melatonin on the transcript levels of target hormonal genes under long-term HL stress, suggesting the identification of delayed response genes under persistent stress conditions. Notably, stress and signalling pathways that are commonly upregulated during short-term HL stress are, in contrast, downregulated during the long-term HL response, suggesting a transition from short-term, nonspecific ROS- and stress-associated responses to long-term acclimation mechanisms [46].

Most MT-hormone interactions in HLs are associated with ABA-mediated responses, which are generally regulated by a negative feedback mechanism. These results are consistent with examples of antagonistic relationships between MT and ABA under stress as a result of redundancy and overlapping effects of both regulators [47]. A similar mode of regulation was predominant for the ethylene genes, although positive cross-talk was also detected, particularly for several transcription factor genes repressed by HL. These transcription factors are involved in special functions that guide cell wall integrity and cuticular wax biosynthesis.

The auxin-related genes were mostly upregulated, suggesting their involvement in the growth-promoting function of melatonin through controlling cell expansion or division. Far fewer MT-affected genes were involved in SA and JA responses, given the participation of these two hormones, primarily in pathogen defense responses. MT reduced the expression of JA- and SA-responsive genes to minimize the plant growth inhibition that underlies defense responses. Transcriptional targets shared by BS, CK and GA were even less numerous, with some, such as *CKG* or *XTH24*, being induced by multiple hormone signals. Within the plant model examined, no intersections were revealed between melatonin and the most recently discovered hormones karrikin and strigalactone.

One significant outcome of this study is the identification of genes whose expression, which is positively or negatively modulated by stress, is further enhanced by MT treatment. These genes may represent MT targets with particular biological validity for discerning specific patterns of MT action. For example, MT upregulated *AT2G38240*, which encodes 2-oxoglutarate (2OG) and Fe(II)-dependent oxygenase superfamily proteins, thereby contributing to a greater reduction in JA accumulation. Increased activation of CK and GA-regulated *CKGs* may further promote plant growth via the activity of downstream targets. On the other hand, enhanced negative regulation may also facilitate stress resistance, as revealed by *GolS2* and *MIPS2*, which are involved in fine-tuning *myo*-inositol homeostasis, or the remorin family gene *AtREM4.2*, whose precise role in the HL stress response remains to be determined. We conclude that these types of MT regulation contribute to greater plant adaptability to stress by enhancing the action of specific hormone-mediated mechanisms.

One such mechanism is implemented by BTB and TAZ domain protein 2 (BT2), which contains BTB, TAZ, and calmodulin binding domains. BT2 is a central component of an interconnected signalling network that detects and responds to multiple and sometimes competing signals, including ABA, SA, JA and auxin. In particular, the repression of *BT2* (*At3g48360*) can induce increased ABA levels, whereas the accumulation of BT2 mRNA potentiates auxin responses [48]. Since MT treatment further increased BT2 transcript levels, melatonin may modulate hormone responses through BT2 by suppressing ABA signalling while enhancing auxin signalling. We speculate that MT-mediated regulation of BT2 contributes to reversible chromatin condensation, modulating the access and binding of specific transcription factors. However, this assumption requires the identification of MT-regulated proteins that interact with BT2, either at the transcriptional or posttranscriptional level. In contrast to BT2, the expression of other BT family members was not significantly responsive to MT treatment, demonstrating target selectivity of MT effects and a lack of functional redundancy in the BT gene family [49].

The study of signal integration between MT and hormones should include identification of the elements of the melatonin signal transduction pathway that interact with hormone signalling or response genes. According to current concepts, melatonin and its derivatives function in receptor-mediated signalling pathways that may involve the PMTR1/CAND2 receptor, which triggers G protein signalling and MAPK cascades [5], or the melatonin-mediated MAPK signalling pathway itself. In particular, melatonin was shown to activate MAPK3/6 in pathogen infection and endoplasmic reticulum stress [50]. In this study, we showed that the expression of several hormone-related genes, such as *CRF6*, *CKG* or *Thi2*, was dependent on the CAND2 receptor pathway, since their transcript levels did not change in the *cand2* mutant in response to MT treatment. However, the induction or repression of other genes (*ABI3*, *ABI4*, and *ERF56*) by MT was not blocked in *cand2*. These controversial results may indicate that the role of the putative MT receptor CAND2 is not universal, implying the existence of additional candidates for its role in MT signal perception or noncanonical signalling through direct interaction of signalling proteins that function in phytohormone signalling pathways [51].

Notably, in our study, MAPK3/6 responded similarly to HL stress with and without MT treatment. However, two other genes, *MAPKKK17* (*AT2G32510*) and *MRKK20* (*AT3G50310*), were significantly regulated by MT treatment. *MAP3K17* was reported to be highly induced by ABA, which was compromised in mutants of the ABA core signalling module. Furthermore, a complete ABA-activated MAPK cascade includes the MAP3Ks MAP3K17/18, the MAP2K MKK3 and the four C group MAPKs, MPK1/2/7/14 [52]. One of these downstream targets of the ABA-triggered MAPK pathway, MKK7, was sensitive to MT treatment in WT plants. Disruption of *CAND2* completely abolished melatonin-dependent activation of MAP3K17 and MKK7, suggesting that the MAP3K17-MKK7 module is an important actor in the MT-triggered MAPK pathway and acts downstream of CAND2. However, further studies involving mutants of the MAP kinase cascade are needed to confirm this signalling circuit.

## 4. Materials and Methods

### 4.1. Plant Material, Growth Conditions and Treatments

Wild-type *A. thaliana* (L.) Heynh. (Columbia and Landsberg erecta) and corresponding mutant lines (*cand 2* (NASC 678658), *abi1-1* (NASC 22), *ahk2/3*, *bri1-6* (NASC 399) were obtained from the Nottingham Arabidopsis Stock Centre or kindly provided by Prof. Tatsuo Kakimoto from Osaka University, Japan. The seeds were sown in Petri dishes on half-strength Murashige and Skoog (MS) media supplemented with 1% sucrose and 0.5% agar, stratified at 4 °C for 3 days and then exposed to 60 μmol m^−2^ s^−1^ white light at 23 °C under a 16 h light/8 h dark cycle. Two-week-old plants were pretreated with 50 μM of melatonin for 72 h and exposed for 24 h under an HPI-T2 2000 W/646 lamp (Philips, Amsterdam, The Netherlands) with a luminous energy flux of 600 μmol m^−2^ s^−1^. A combination of air flow and water flow cooling was used to minimize the thermal impact of the lamp. The control plants were kept under growing conditions. The plant tissue was frozen in liquid nitrogen and stored at −80 °C for further analysis (Figure 1).

### 4.2. Stress Tolerance Tests

The secondary products of membrane lipid peroxidation (TBARs) were determined by a reaction with thiobarbituric acid, as described by Heath and Packer [53]. The measurement was performed on a spectrophotometer (Pharmacia Biotech Ultrospec 2000, London, UK) at wavelengths of 532 and 600 nm. The amount of TBARs was calculated in μmol/g FW.

Electrolyte leakage was measured with a Seven2Go S3 instrument equipped with an InLab 731-ISM electrode (Mettler Toledo, Columbia, MD, USA) and calculated as the percentage of the conductivity before boiling over that after boiling [54].

Hydrogen peroxide (H_2_O_2_) was detected via the titanium chloride method [55]. Plant tissue (100 mg) was homogenized in 200 μL of chilled 100% acetone and centrifuged for 10 min at 10,000× *g*. A total of 160 μL of the supernatant was transferred to clean tubes and mixed with 40 μL of 20% TiCl_4_ solution, followed by 40 μL of concentrated NH_4_OH solution. The precipitated titanium peroxide was washed with 100% acetone and resuspended in 2 N H_2_SO_4_ solution. The absorbance was measured at 415 nm, and the H_2_O_2_ content was calculated via a calibration curve of H_2_O_2_.

The rate of O_2_^⋅−^ generation was assessed as described by Nahar et al. [56]. The plant material was homogenized in 50 mM phosphate buffer solution (pH 7.8) and centrifuged at 5000× *g* for 10 min. The supernatant was mixed with extraction buffer and 10 mM of hydroxylamine hydrochloride and incubated at 25 °C for 20 min. Then, 17 mM of sulfanilamide and 7 mM of naphthylamine were added, and the mixture was incubated again at 25 °C for 20 min. The optical density of the solution was measured at 530 nm. The rate of O_2_^⋅−^ generation was calculated via the standard curve of NaNO_2_.

SOD activity was assessed according to the protocol of Giannopolitis and Ries [57]. One hundred milligrams of plant tissue was homogenized with phosphate buffer. The homogenate was centrifuged for 15 min at 12,000× *g* at 4 °C. A solution of 0.44% riboflavin was added to a mixture containing the extract of the material and the reaction mixture (phosphate buffer, methionine, NBT, and Triton at a ratio of 3:1 0.75), and the mixture was immediately placed under illumination at 120 μmol m^−2^ s^−1^ for 30 min. A mixture containing no enzyme extract served as a light control. Measurements were performed at 560 nm. The activity was determined in arbitrary units after recalculation relative to the light control.

The activity of guaiacol-dependent peroxidase was determined according to the methods of Shevyakova et al. [58]. The reaction mixture contained 1 part supernatant, 20 parts 0.066 M potassium phosphate buffer (pH 7.4), 4 parts 7 mM of guaiacol, and 4 parts µL 0.01 M H_2_O_2_. The absorbance of the solution was measured at 470 nm.

### 4.3. Determination of Pigments and Fluorometry

Chlorophyll and carotenoids were extracted and quantified as described by Lichtenthaler [59]. Pigments were extracted from plant rosette leaves with 80% (*v*/*v*) acetone. The concentrations of total chlorophyll (Chl a + *b*) and carotenoids were determined by examining the absorbance at 440, 649, and 665 nm in the centrifuged supernatant.

Chlorophyll fluorescence parameters (variable fluorescence to maximum fluorescence (Fv/Fm), the effective quantum yield of PSII (Φ_PSII_) and NPQ non-photochemical quenching) were measured with a DUAL-PAM101 (Heinz Walz GmbH, Effeltrich, Germany) fluorometer as described by Kozuleva et al. [60]. Measurements were taken for three to six leaves from three plants. The following parameters were determined: light, 460 nm; pulses, 9 µmol m^−2^ s^−1^; 500 ms; 635 nm; 4000 µmol m^−2^ s^−1^; and actinic light, 635 nm; and 37 µmol m^−2^ s^−1^. The dark incubation time for the measurements was 10 min.

### 4.4. RNA Extraction, Library Construction, and RNA Sequencing

Total RNA was extracted from all the samples via a HiPure Total RNA Kit (Magen, Guangzhou, China). Extractions were performed from three biological replicates, each representing a mixture of five plants. The RNA concentration and purity were quantified via a Bioanalyzer 2100 (Agilent, Santa Clara, CA, USA). A total of 700 ng of total RNA with a RIN ≥7 was used to generate the library via the NEBNext^®^ Poly(A) mRNA Magnetic Isolation Module and the NEBNext^®^ Ultra II™ Directional RNA Library Prep Kit for Illumina (New England Biolabs, Ipswich, MA, USA) according to the manufacturer’s instructions. Paired-end sequencing was performed on a NovaSeq 6000 instrument (Illumina, San Diego, CA, USA) following the manufacturer’s recommendations.

The raw sequences were transformed into clean reads with the parameters “ILLUMINACLIP::2:30:10 LEADING:20 TRAILING:20 SLIDINGWINDOW:4:15 MINLEN:30” in paired-end mode after data processing via Trimmomatic v. 0.36 [61]. The resulting high-quality clean reads were mapped to the reference genome of *A. thaliana* (version TAIR10.1), and reads per gene were counted via STAR v. 2.7.10b [62] with the parameters “--sjdbGTFfeatureExon exon --sjdbGTFtagExonParentTranscript transcript_id --sjdbGTFtagExonParentGene gene_id --quantMode GeneCounts”.

### 4.5. Differential Expression and Ontology Enrichment Analyses

Differentially expressed genes (DEGs) were identified via the “DESeq2” 1.48.0 R package [63] with thresholds of adjusted *p* value (FDR) < 0.05 and fold change > 2. The functional enrichment analysis included GO and KEGG term enrichment. Overrepresented DEGs were analysed via the Database for Annotation, Visualization, and Integrated Discovery (DAVID) online tool. FDR-corrected *p* values < 0.05 and fold enrichment > 2 were used as thresholds to identify significantly enriched terms [64,65]. The sequencing data are given in Supplemental Tables S1–S3.

#### Quantitative Real-Time Polymerase Chain 260 (qRT–PCR)

Total RNA was extracted from frozen leaves via a HiPure Total RNA Kit (Magen, China). The isolated RNA (2 μg) served as a template for cDNA synthesis via RevertAid reverse transcriptase Thermo Fisher Scientific, Waltham, MA, USA) and a mixture of oligo-(dT)15 and random primers (DNA synthesis, Moscow, Russia). The primer pairs used for qRT–PCR are listed in Supplemental Table S4. Quantitative real-time PCR was performed in a LightCycler 96 (Roche, Rotkreuz, Switzerland) with hot start SYBR Green I technology. The standard thermal profile for all PCRs included the following steps: 95 °C for 10 min; 40 cycles of 95 °C for 15 s, 58 °C for 15 s, and 72 °C for 20 s; and melting curve analysis. The relative transcript abundance of the tested genes was calculated via the 2^−ΔΔCt^ method and normalized to the transcript level of the nuclear-encoded polyubiquitin *UBQ10* gene, which was used as internal standard.

### 4.6. Statistical Data Processing

The experiments were performed in three biological replicates, and the results were averaged. The significance of differences was estimated via one-way analysis of variance (ANOVA) followed by Tukey’s method via an online calculator (https://astatsa.com/OneWay_Anova_with_TukeyHSD/ accessed on 1 October 2025) and Student’s *t* test.

## 5. Conclusions

Phytohormones, as universal regulators of plant growth, form a complex web of interactions so that all hormone treatments may trigger a ‘‘domino effect,’’ resetting many systems within the plant [66]. This work aimed to identify hormone-responsive genes affected by melatonin treatment under long-term HL stress. By means of RNA-seq analysis, we identified more than 70 genes associated with hormone metabolism, hormone signalling, and hormone response. The genes that were targeted by melatonin under high light conditions included those encoding receptors, kinases, and transcription factors, as well as genes associated with Ca signalling, transport, ubiquitination and secondary metabolism. As a stress-alleviating agent, MT suppressed growth-inhibiting genes and activated growth-promoting genes. However, changes in the expression of certain genes induced by stress were even further strengthened, suggesting the involvement of MT in specific stress-related reactions. These genes are of particular interest for deciphering the unique mechanisms of MT action and for use in biotechnological programs.

Our study revealed novel hormonal genes regulated by MT and raised important questions about the underlying signalling mechanisms involved. Analysis of the MT signal transduction mutant revealed that the MT-dependent responses of hormonal genes were partially dependent on the CAND receptor. In particular, CAND2 may activate the MAP3K17-MKK7 module in ABA-dependent responses and trigger the expression of CK signalling genes. However, the role of CAND2 in MT signal transduction is not comprehensive, suggesting the existence of alternative candidates for MT perception or the nontranscriptional interaction of signalling proteins.

## Data Availability

Data are contained within the article and Supplementary Materials.

## Supplementary Materials

The following supporting information can be downloaded at: https://www.mdpi.com/article/10.3390/ijms262110531/s1.
