# Supplementary material for: Melatonin at Crossroads with Phytohormones: Interactions Under High Light Stress
Source: Int J Mol Sci. 2025 Oct 29;26(21):10531. doi: 10.3390/ijms262110531 (PMC12607704; doi:10.3390/ijms262110531)
Supplement: Supplementary file 1 [file ijms-26-10531-s001.zip › Figure S1. Spectra of lamps.pdf]

A

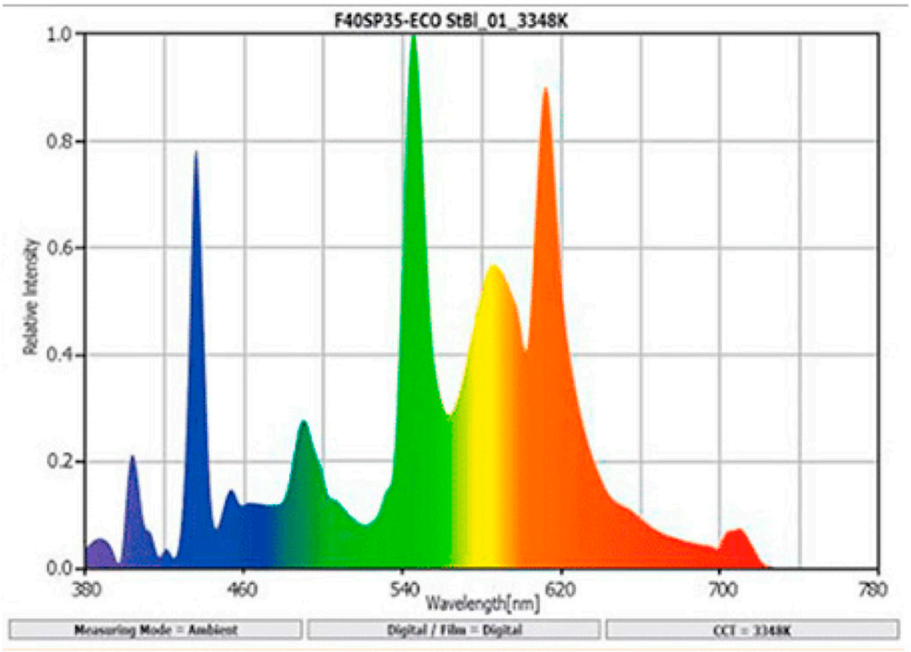

B

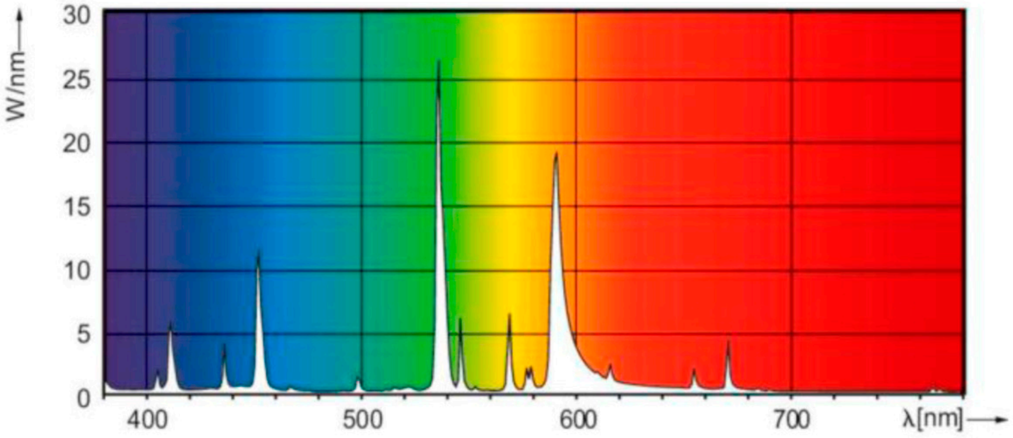

Figure S1. Spectra of lamps. A – Growing conditions, F40SP35/ECO (GE Ecolux, USA), B – Stress conditions, HPI-T2 2000 W/646 (Philips, Netherlands).
